# Chinese Herbal Formula Xuefu Zhuyu for Tension-Type Headache with *Qi*-Stagnation and Blood-Stasis Pattern (CheruXTH): Study Protocol for a Randomized Controlled Trial

**DOI:** 10.1155/2020/5653169

**Published:** 2020-09-11

**Authors:** Li Zhou, Zhe Zhang, Geng Li, Shaojun Liao, Hongfei Zhou, Pengqin Wang, Guanghui Liu, Li Bai, Zehuai Wen

**Affiliations:** ^1^Second Clinical Medical College, Guangzhou University of Chinese Medicine, Guangzhou, Guangdong, China; ^2^Second Affiliated Hospital of Guangzhou University of Chinese Medicine, Guangzhou, Guangdong, China; ^3^Key Unit of Methodology in Clinical Research, Guangdong Provincial Hospital of Chinese Medicine, Guangzhou, Guangdong, China; ^4^Liaoning University of Traditional Chinese Medicine, Shenyang, Liaoning, China; ^5^Department of Neurology, Affiliated Hospital of Liaoning University of Traditional Chinese Medicine, Shenyang, Liaoning, China; ^6^Guangdong Provincial Key Laboratory of Clinical Research on Traditional Chinese Medicine Syndrome, Guangzhou, Guangdong, China; ^7^State Key Laboratory of Dampness Syndrome of Chinese Medicine, Second Affiliated Hospital of Guangzhou University of Chinese Medicine, Guangzhou, Guangdong, China

## Abstract

**Background:**

Tension-type headache (TTH) is the most common headache disorder. Current treatments for TTH have been reported to be associated with insufficient long-term benefits and unwanted adverse events (AEs). The Chinese herbal formula Xuefu Zhuyu (XFZY) has been utilized in TTH treatment, but the evidence supporting its efficacy remains unclear. This study will evaluate the efficacy and safety of XFZY for TTH.

**Methods:**

This multicenter, double-blind, randomized, placebo-controlled trial will be undertaken in China. A total of 174 eligible participants will be randomly assigned to either an XFZY group or a placebo group (20 ml each dose, three times daily for 4 weeks) at a ratio of 1 : 1. The primary outcome is the change in mean headache intensity measured by a 10 cm visual analogue scale (VAS). Secondary outcomes include the area-under-the headache curve (AUC), headache frequency, rescue medication use, *qi-stagnation and blood-stasis* pattern measurement, quality of life measured by the EuroQol-5-Dimensions-5-Level (EQ-5D-5L), global evaluation of medication, and health economic indexes. *Discussion*. The results of the study are expected to provide evidence of high methodological and reporting quality on the efficacy and safety of XFZY for TTH. This trail is registered with ChiCTR1900026716 (registered on 19 October, 2019).

## 1. Introduction

Tension-type headache (TTH) is the most prevalent headache disorder [1, 2] and the second most common disease in the 2010 Global Burden of Disease Survey [3, 4]. It has a lifetime prevalence ranging from 30% to 78% [5]. In China, the years lived with disability (YLDS) for TTH are second only to migraine among headache disorders [2]. This has immense social and economic costs. Overall absence rates for subjects with frequent TTH are on the rise, despite not increasing for migraines [6]. Hence, it is one of the most costly disorders to society [7]. In order to improve Chinese citizens' quality of life, treating TTH should be a national priority [2].

Simple analgesics and nonsteroidal anti-inflammatory drugs (NSAIDS) are recommended for episodic TTH (ETTH) by the European Federation of Neurological Societies (EFNS) [8] and the British Association for the Study of Headache (BASH) [9]. They have been shown to be to be effective for ETTH [10–12] but not for chronic TTH (CTTH). As headache frequency increases, so does the risk of medication overuse, especially in CTTH. The tricyclic antidepressant amitriptyline is the primary choice for the prophylactic treatment of CTTH [8]. However, the effect of these drugs is not satisfactory because of insufficient effect or side effects [13, 14]. The combination of caffeine with simple analgesics is more effective for the treatment of ETTH than simple analgesics alone, but at the cost of an increased frequency of side effects [15, 16]. Hence, combination analgesics containing caffeine are treated as the second choice for ETTH by EFNS guideline [8]. Opioids should not be used because of drug resistance, dependence, and toxicity [8]. Furthermore, the use of these drugs also increases the risk of forming medication-overuse headache (MOH) [17]. Other treatments have also been applied in clinic, including Chinese medicine (CM) therapies, cognitive behavioral therapy, physical therapy, stress management, and acupuncture, but the evidence for these treatments is limited or inconsistent [18, 19]. Therefore, more research is needed to explore new medications.

Chinese herbal medicine (CHM) has a long history in China and other regions of Asia. It is the main form of CM and could provide further options for headache treatment. The Chinese herbal formula Xuefu Zhuyu (XFZY) is the representative, classical CHM prescription for the treatment of headache. It has been widely used in the treatment of headache with *Qi-stagnation and Blood-stasis* pattern (QBS) (also called Zheng in CM). XFZY oral liquid is a patented product, which has been approved by the China Food and Drug Administration in 2002 (Ref No. Z10950063) [20]. It is an improved dosage form of XFZY decoction [21], which strictly follows the original prescription and dosage of XFZY decoction. Hence, they have the same function in promoting and activating the flow of *qi* and *blood* to treat QBS. This treatment has been utilized since the Qing Dynasty. XFZY consists of Bupleuri Radix (chaihu), Angelicae Sinensis Radix (danggui), Rehmanniae Radix (shengdihuang), Paeoniae Radix Rubra (chishao), Carthami Flos (honghua), Persicae Semen (taoren), Aurantii Fruxtus (zhiqiao), Glycyrrhizae Radix et Rhizoma (gancao), Chuangxiong Rhizoma (chuanxiong), Achyranthis Bidentatae Radix (niuxi), and Platycodonis Radix (jiegeng). A document to describe the components of the XFZY and pharmaceutical production processes were offered in supplementary file S1. Experimental studies have revealed that XFZY has an anticoagulating effect and improves microcirculation by reducing blood viscosity [22–25]. Clinical studies have shown that XFZY is effective in treating headache disorders, especially in migraine [26, 27], but the evidence of XFZY for TTH is absent. Therefore, the aim of this trial is to investigate whether the efficacy and safety of XFZY for TTH patients with QBS is superior to placebo.

## 2. Methods

### 2.1. Study Design and Setting

**Figure 1:**
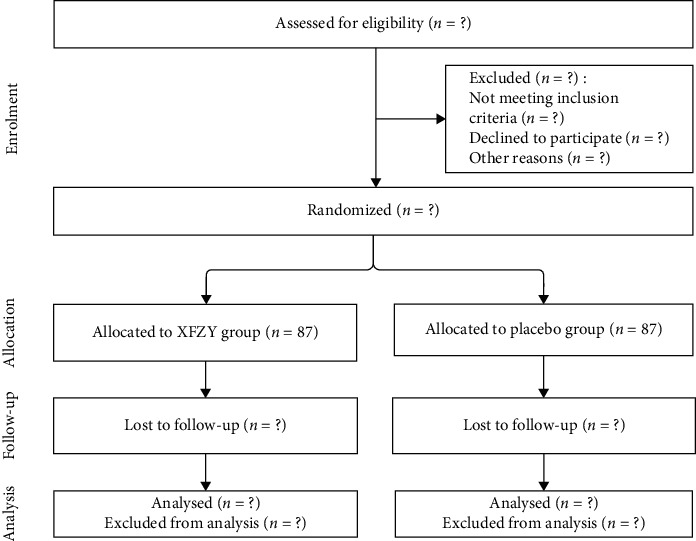
Study flow chart.

This is a multicenter, double-blinded, randomized, placebo-controlled trial that will be conducted in six hospitals located in different regions of China. The screening condition of the center is whether the hospital is a state clinical research base or a clinical trial institution and can complete the observation of at least 20 patients. Six centers are listed below: (1) Affiliated Hospital of Liaoning University of Traditional Chinese Medicine; (2) Second Affiliated Hospital of Guangzhou University of Chinese medicine; (3) Second Hospital Affiliated to Liaoning University Traditional Chinese Medicine; (4) First Hospital of China Medical University; (5) Shenyang 10th people's Hospital; and (6) Affiliated Hospital of Inner Mongolia University for the Nationalities. Eligible participants will be randomly assigned to either an experimental group or a placebo group at a ratio of 1 : 1 (Figure 1). The trial's protocol reporting follows the Standard Protocol Items for Clinical Trials with Traditional Chinese Medicine (SPIRIT-TCM) (Supplementary file S2) [28].

### 2.2. Participants

We will recruit potential eligible participants via newspaper advertisements, notice boards, and outpatient visits, and also pay attention to whether the recruitment and enrollment progress reached the target sample size. Patients who meet the inclusion criteria and sign informed consent forms will enter the screening period; otherwise, they will be excluded before randomization.

Patients in each center will be diagnosed and differentiated by a senior neurologist and will be observed by a neurologist at a specialist clinic. TTH diagnostic criteria include (1) Western medicine diagnosis based on the criteria of the International Classification of Headache Diseases-3 (ICHD-3) published by the International Headache Society (IHS) in 2018 [5] and (2) pattern diagnosis according to a validated QBS diagnostic scale [29] (Table 1).

Eligibility criteria are as follows: (1) TTH must have occurred for at least 1 year, at least 2 days per month (unlimited frequency), and at least one TTH attack needs to have lasted over 4 hours; (2) a visual analogue scale (VAS) score ≥3 cm; (3) the first onset of TTH need to have happened before the age of 50 years; (4) aged 18–65 years; and (5) CM pattern differentiation is QBS.

Exclusion criteria are (1) concurrent migraine; (2) ailment diagnosed as secondary headache by computed tomography (CT) and transcranial Doppler (TCD) within the previous 6 months; (3) having serious primary diseases such as those of the cardiovascular, liver, kidney or hematopoietic system, and psychosis (schizophrenia, epilepsy, alcoholism, and anorexia) or major neuropsychiatric diseases; (4) labs showing increase in alanine aminotransferase (ALT) or aspartate aminotransferase (AST) over twice the upper limit of the normal range or an increase in creatinine over 1.5 times of the upper limit of the normal range; (5) suffering from other severe pain-inducing diseases, such as cancer; (6) patients with a Self-rating Anxiety Scale (SAS) greater than 59 or Self-rating Depression Scale (SDS) greater than 62; (7) potential allergy to XFZY; (8) women who are either pregnant, lactating, or planning to get pregnant; and (9) participating in other clinical trials within the previous month.

### 2.3. Randomization

A block randomization sequence stratified by centers is generated by SAS 9.2 (SAS Institute Inc., Cary, USA) and performed by the Institute of Basic Research in Clinical Medicine (IBRCM), China Academy of Chinese Medical Sciences, through an interactive web response system. When eligible patients who have signed informed consent forms are enrolled, an independent researcher will log into the system to obtain their allocation results. The randomization results will be kept confidential and maintained by IBRCM. All patients and researchers, including investigators, outcome assessors, statisticians, and other staff involved in the trial will be unaware of the grouping and treatment during the study period.

### 2.4. Blinding

The investigators, participants, research assistants, and statisticians will be blinded to the allocated treatment. Both the XFZY and the matched placebo are manufactured by Jilin Aodong Yanbian Pharmaceutical Co., Ltd. (Dunhua, Jilin, China). The placebo is identical in color, size, dosage form, smell, and taste to XFZY. Both XFZY and the placebo will be labeled and packaged based on a randomization schedule from the clinical research department at Jilin Aodong Yanbian Pharmaceutical Co., Ltd., under the supervision of IBRCM. The blinding code will not be revealed until the end of the trial, unless a patient has a serious adverse event (SAE) or emergency which necessitates knowing what medications have been taken.

### 2.5. Interventions

#### 2.5.1. Experimental Group

In the experimental group, participants will take 20 ml XFZY orally, 3 times a day, for 4 weeks. After treatment, an 8-week follow-up will be conducted. XFZY oral liquid will be manufactured in strict accordance with the standards of the Chinese Pharmacopoeia (2015) [30], and the process strictly abides by the Good Manufacturing Practice (GMP). XFZY will be manufactured by Jilin Aodong Pharmaceutical Group Co., Ltd., in line with the requirements of GMP.

#### 2.5.2. Placebo Group

Placebo was selected as the comparator to assess the efficacy of XFZY owing to substantial placebo effect in the treatment of headache [31]. Participants in the placebo group will also be given 20 ml of the placebo, 3 times a day for 4 weeks. The follow-up period is the same as that of the experimental group. The placebo is made from honey, white granulated sugar, fried brown sugar, bitterant, natural edible pigments, food antiseptic, and thimbleful ginseng essence, to mimic the flavor of Chinese herbs. It also meets the hygiene inspection requirements. The placebo will also be provided by Jilin Aodong Pharmaceutical Group Co., Ltd., in line with the requirements of GMP.

#### 2.5.3. Rescue and Concomitant Treatment

Participants in both groups will be consistent in usage, dosage, and trial period. The painkiller (acetaminophen) will be allowed to relieve symptoms, if unbearable pain remains after taking the study drugs for two hours. During the treatment period, use of CM decoction, other CM products, acupuncture, massage, or cupping therapy with the CM function of moving *qi* and/or activating *blood* will be forbidden.

### 2.6. Drug Compliance

The drug compliance is measured by the tablet count. The patients are asked to return the packaging from the drugs they have already taken at each follow-up visit, throughout the study period. The formula is based on the amount of medication taken divided by the total amount of medication to be taken, and ≥80% will be considered high compliance.

### 2.7. Outcome Measures

#### 2.7.1. Primary Outcome

The primary outcome is the change in mean headache intensity from the baseline to the end of the 12th week. Headache intensity will be measured along a 10 cm visual analogue scale (VAS), with “none” and “very severe” at the extremes of the spectrum.

#### 2.7.2. Secondary Outcomes

The secondary outcomes are the area-under-the-headache curve (AUC), calculated by the sum of the daily headache duration (hours) multiplied by the daily headache intensity for each observed period and, then, divided by the observation period; headache frequency, defined as a ≥50% reduction by number of days with a headache or attacks per evaluation period; headache hours in each observed period; rescue medication use for unbearable pain 2 hours after taking the drugs; QBS pattern measurement by a qualified CM physician; quality of life measured by the EuroQol-5-Dimensions-5-Level (EQ-5D-5L) granted by the EuroQol Group [32, 33]; global evaluation of medication assessed by a simple verbal scale; health economic indexes calculated by the cost of registration, laboratory testing, drugs (other than the experimental drug), hospital admission, transportation, average monthly income, and costs incurred by family caregivers. Participants will be asked to complete a headache diary throughout the trial. The primary outcome and the first four secondary outcomes will be extracted from the headache diary.  Table 2 shows the timing for all outcome measures in the study.

### 2.8. Safety Assessment

Any adverse events (AEs) that occur during the study period will be reported to the research assistants, and the causality between AE and drug will be assessed according to the WHO Uppsala Monitoring Center System for Standardized Case Causality Assessment [34]. Laboratory examinations including the blood routine test, urine routine test, liver function, kidney function, coagulation function, and electrocardiogram will be performed at the baseline and the fourth week. Attention is paid to the abnormal changes of laboratory test results, they are recorded as an AE if they have clinical significance, and the causality with the study drug is assessed.

An independent Data and Safety Monitoring Committee (DSMC) will also evaluate the safety data which will be requested during the trial. Severe AEs (SAEs) or severe adverse reactions (SARs) are defined according to the International Council for Harmonization of Technical Requirements for Pharmaceuticals for Human Use (ICH) guidelines [35]. They must be reported to the DSMC, the GPHCM ethnics committee, and the research team within 24 h. AE or SAE details such as occurrence, severity, management, and causality to the intervention will be recorded on electric case report forms (eCRFs). In emergency situations, physicians will be consulted, and the blinding will be broken if necessary.

### 2.9. Sample Size

The sample size was calculated based on a mean change of 4.46 with a standard deviation (SD) of 1.5 scores in the XFZY group [36], a mean change of 2.14 with an SD of 1.5 scores in the placebo group [37], and the minimal clinically important difference set to 1.7 [38]. A sample size of 87 patients per group was determined to provide 80% power to achieve statistical significance at the 5% 2-sided level and a superiority margin of 1.7 for comparisons of XFZY and placebo for the primary outcome. This calculation allowed for a 15% rate of withdrawal and loss to follow-up.

### 2.10. Statistical Analysis

The statistical analysis will be performed on the basis of a pre-established statistical analysis plan. Data analysis will be based on the intent-to-treat (ITT) and per protocol (PP) principles and will be conducted by independent qualified statisticians blinded to treatment allocation. Two-tailed *p* values less than 0.05 will be considered statistically significant. Missing data will be replaced by the multiple imputation method. All analyses will be conducted with SPSS version 18.0 (IBM SPSS Inc, Armonk, New York, USA) or SAS 9.2 (SAS Institute Inc., Cary, USA). Continuous variables will be expressed as mean ± SD if data are normally distributed. Data not normally distributed will be presented using the median and interquartile range (IQR). Categorical variables will be expressed as counts and percentages.

For primary and secondary outcomes, a full analysis set including participants with at least one treatment and outcome assessment and a perprotocol analysis set containing 4-week treatment,will be employed to compare the changes between groups with an independent *t*-test, respectively; repeated measures of analysis of variance or a liner mixed model will be applied to analyze the changes measured at different time points. Sex, age, course of disease, and center, as well as other covariates will be considered in the statistical model.

For safety assessments, a safety analysis set with all subjects who took the drug at least once will be adopted to detect whether the AE proportions are significant between the two groups. This will be performed with either a *χ*^2^ test or Fisher's exact test. All AEs will be coded based on the Chinese version of the Medical Dictionary for Regulatory Activities (MedDRA).

### 2.11. Data Management and Trial Monitoring

All involved investigators from different centers will be trained uniformly before the study begins, in order to maintain protocol implementation consistency. They will also be given standard operation procedures (SOPs). The electric data capture (EDC) system developed by IBRCM will be used to collect study data. This will allow for auto logical check according to preset procedures. Data managers also can send queries to investigators through the system for manual verification. Then, feedback from investigators is sent to data managers to ensure that the data can be uploaded to the system timely, accurately, and completely, so that the principal investigator can monitor research progress in real time. Data monitoring will include data traceability, recruitment, randomization, and compliance. It will be conducted regularly with SOPs by an independent department from Jilin Aodong Yanbian Pharmaceutical Co., Ltd. (Jilin, China) called the clinical data management team. Audits will be executed regularly by IBRCM at the China Academy of Chinese Medical Sciences. Meanwhile, the trial will be received the supervision and inspection of the Ministry of Science and Technology and the Hospital Scientific Research Department.

### 2.12. Ethics and Dissemination

This trial protocol has been approved by the Ethics Committee at Guangdong Provincial Hospital of Chinese Medicine (GPHCM) (Approval No. BF2019-175-01) and other ethics committees at each center. Written informed consent must be obtained from all participants. The study will be conducted in compliance with the Declaration of Helsinki [39] and good clinical practice guidelines [40]. The results will be published in a peer-reviewed journal and a PhD dissertation and will be presented at conferences.

## 3. Discussion

To our knowledge, this is the first study to investigate the efficacy and safety of XFZY for TTH, compared to a placebo treatment. Although existing pharmacological and nonpharmacological treatments have been shown to be helpful [11, 41–43], the evidence available for treatment efficacy is limited to small magnitude or adverse events. Hence, new pharmacologic treatments are expected.

Both ETTH and CTTH patients are target populations of this study. However, they are categorized differently according to ICHD-3 criteria and have variant pathogenesis [44, 45]. Yet, the two diseases can be identified by the same CM pattern and are judged by similar signs and symptoms. QBS is a common TTH pattern. XFZY is a classical formula that promotes the flow of *qi* and *blood* in CM. Hence, we will conduct a randomized, double-blinded, placebo-controlled trial to evaluate the effect of XFZY for TTH with QBS.

Patient-centered outcomes are the focus of this study. This is in contrast to previous studies which have concentrated on rapid relief and being pain-free after 2 h, as recommended by HIS [31]. VAS and EQ-5D-5L are reported by the subjects, and the former is the primary outcome for evaluating headache intensity, while the latter measures the quality of life. The long-term effect deserves more attention than the frequent occurrence of headache. Therefore, our study period includes 4 weeks of treatment and 8 weeks of follow-up, in order to observe the long-term response.

TTH has immense public health costs, both in China and around the world, owing to its high prevalence among the general population, especially with regard to indirect costs (i.e., lost work and leisure time). As such, the economic costs are increasing. Data from a cross-sectional survey has shown mean annual costs of €303 per capita for tension-type headache in Europe [46]. Among them, the direct cost is €25, less than CNY 468 per person in China [47]. In order to assess costs and cost-effectiveness for patients taking XFZY compared with patients receiving placebo, we will calculate the health economic index as one of the outcome measures.

In conclusion, the results of this study are expected to provide evidence of high methodological quality on the efficacy and safety of XFZY for TTH.

## Figures and Tables

**Table 1:** QBS diagnosis scale.

Symptoms/signs	Yes	No	Score
Pain	9	0	
Irritability/depression	16	0	
Distending pain	2	0	
Scurry pain	6	0	
Chest distress	0.5	0	
Lumps in body	7	0	
Petechia in the tongue	4	0	
Purplish tongue	1	0	
Unsmooth pulse	4	0	
Deep pulse	2	0	
Total score If ≥20 points, diagnosed as QBS			

Notes: pain includes stomachache, abdominal pain, low back pain, dysmenorrhea, breast pain, and limb pain. QBS: *Qi*-stagnation and Blood-stasis pattern.

**Table 2:** Outcome measures.

Domain	Measure	Time (weeks)
*Primary*		
Mean pain intensity of headache change	10 cm VAS with “none” and “very severe” at either end	0, 1, 2, 4, 8, 12
Secondary		
AUC	Calculated by the sum of the daily headache duration (hours) multiplied by the daily headache intensity in each observed period and, then, divided by the observation period	0, 1, 2, 4, 8, 12
Headache frequency	Defined as a ≥50% reduction by the number of days with a headache or attacks per evaluation period; headache hours for each observed period	0, 1, 2, 4, 8, 12
Headache hours	The total hours in each observed period	0, 1, 2, 4, 8, 12
Rescue medication use	Records the number of participants using the rescue medication and the total dosage per week	0, 1, 2, 4, 8, 12
QBS measurement	Evaluated by a qualified CM physician	0, 1, 2, 4, 8, 12
Quality of life	EQ-5D-5L	0, 4, 12
Global evaluation of medication	A simple verbal scale: very poor, poor, neutral, good, very good	4
Health economic indexes	Registration costs, laboratory tests, drugs (other than the experimental drug), hospital admission, transportation, average monthly income, costs incurred by family caregivers	0, 1, 2, 4

VAS: visual analogue scale, AUC: area-under-the-headache curve. EQ-5D-5L: EuroQol-5-Dimensions-5-Level.

## Data Availability

The datasets used and analyzed in the current study are available from the corresponding author on reasonable request after the study completion. The date and version identifier of the protocol are V1.0/20190920.

## Supplementary Materials

Supplementary Materials S1: the dose and scientific names of all ingredients in XFZY oral liquid. S2: SPIRIT-TCM Extension 2018 checklist.
