# Supplementary material for: Chinese Herbal Formula Xuefu Zhuyu for Tension-Type Headache with Qi-Stagnation and Blood-Stasis Pattern (CheruXTH): Study Protocol for a Randomized Controlled Trial
Source: Evid Based Complement Alternat Med. 2020 Sep 11;2020:5653169. doi: 10.1155/2020/5653169 (PMC7502128; doi:10.1155/2020/5653169)
Supplement: Supplementary Materials — S1: the dose and scientific names of all ingredients in XFZY oral liquid. S2: SPIRIT-TCM Extension 2018 checklist. [file 5653169.f1.zip › 5653169.f1/Supplemental file 2--SPIRIT-TCM Extension 2018 checklist.docx]

**Appendix 2 SPIRIT-TCM Extension 2018 checklist**

| **Item** | **SPIRIT 2013 statement** | | **page** | **SPIRIT-TCM Extension 2018** | | **Reported on page #** |
| --- | --- | --- | --- | --- | --- | --- |
|  | **Administrative information** | | | | | |
| Title | 1. Descriptive title identifying the study design, population, interventions, and, if applicable, trial acronym | |  | 1a. Specify the patient population in terms of 1) a WM-defined disease, 2) a WM-defined disease with a specific TCM Pattern, or 3) a TCM Pattern. | | 1 |
|  |  |  |  | 1b. Specify the intervention, in terms of 1) CHM formula, 2) acupuncture, 3) moxibustion, or 4) other TCM therapy (ies). | | 1 |
| Trial registration | 2a. Trial identifier and registry name. If not yet registered, name of intended registry | |  |  | | 2 |
|  | 2b. All items from the World Health Organization Trial Registration Data Set | |  |  | | Please see the following register webpage:  http://www.chictr.org.cn/showproj.aspx?proj=44489 |
| Protocol version | 3. Date and version identifier | |  |  | | Please see the supplementary material: ethics approval |
| Funding | 4. Sources and types of financial, material, and other support | |  |  | | 15 |
| Roles and responsibilities | 5a. Names, affiliations, and roles of protocol contributors | |  |  | | 1, 16 |
|  | 5b. Name and contact information for the trial sponsor | |  |  | | 1 |
|  | 5c. Role of study sponsor and funders, if any, in study design; collection, management, analysis, and interpretation of data; writing of the report; and the decision to submit the report for publication, including whether they will have ultimate authority over any of these activities | |  |  | | 15 |
|  | 5d. Composition, roles, and responsibilities of the coordinating centre, steering committee, endpoint adjudication committee, data management team, and other individuals or groups overseeing the trial, if applicable (see Item 21a for data monitoring committee) | |  |  | | 13 |
| Introduction | | | | | | |
| Background and rationale | | 6a. Description of research question and justification for undertaking the trial, including summary of relevant studies (published and unpublished) examining benefits and harms for each intervention |  | 6a.1 Provide the background and rationale of the research question with TCM theory. | | 3 |
|  |  |  |  | 6a.2 Describe the rationale of the utilized TCM interventions with references. | | 3-4 |
|  |  |  |  | 6a.3 Provide the rationale of adding experimental TCM interventions if WM intervention is used as basic or combined remedy. If possible, the potential interaction between WM intervention and TCM intervention (especially for CHM) should also be explained with related reference(s). | | 3-4 |
|  |  | 6b. Explanation for choice of comparators |  | 6b. Describe the rationale and principle(s) for selecting comparators corresponding to certain interventions (i.e. CHM formula, acupuncture, moxibustion or other TCM interventions), considering 1) comparable with tested intervention; 2) success of blinding. | | 8 |
| Objectives | | 7. Specific objectives or hypotheses |  | 7. State the objectives or hypotheses regarding the specific TCM intervention for 1) a WM-defined disease, 2) a WM-defined disease with a specific TCM Pattern or 3) a TCM Pattern. | | 4 |
| Trial design | | 8. Description of trial design including type of trial (eg, parallel group, crossover, factorial, single group), allocation ratio, and framework (eg, superiority, equivalence, noninferiority, exploratory) |  |  | | 4, 12 |
| **Methods** | | | | | | |
| **Participants, interventions, and outcomes** | | | | | | |
| Study setting | | 9. Description of study settings (eg, community clinic, academic hospital) and list of countries where data will be collected. Reference to where list of study sites can be obtained |  |  | | 4-5 |
| Eligibility criteria | | 10. Inclusion and exclusion criteria for participants. If applicable, eligibility criteria for study centres and individuals who will perform the interventions (eg, surgeons, psychotherapists) |  | 10a. State whether participants with a specific TCM Pattern will be recruited, in terms of 1) diagnostic criteria, and 2) inclusion and exclusion criteria. All criteria utilized should be universally recognized, or reference(s) where detailed explanation can be found should be given. | | 6-7 |
|  |  |  |  | 10b. Descriptions of the roles, qualifications and other relevant experience of the researchers (e.g. participant screeners, care providers, outcome assessors, data analysts) in TCM research are recommended. | | 6 |
|  |  |  |  | 10c. Descriptions of the qualification and relevant experience of study center(s) involved in a TCM trial are recommended. | | 4 |
| Interventions | | 11a. Interventions for each group with sufficient detail to allow replication, including how and when they will be administered |  | 11a.1 Interventions for the experimental group(s) with sufficient detail to allow replication. | | 8 |
|  | |  |  | 11a.1A. CHM formulae  For fixed CHM formulae  1. Name, source and dosage form (e.g. decoction, granules, powder, pills).  2. Name, source, processing method and dosage of each medical substance. Name of all substances should be presented in at least two types of languages: Chinese (Pinyin), Latin or English. Names of the parts of the substances used should be specified.  3. Authentication method of each ingredient, and how, when, where, by whom it will be conducted.  4. Production method of the formula.  5. Quality control of each ingredient and the whole formula.  6. Safety assessment of the formula, containing heavy metals and toxic elements test, pesticide residue test, microbial limit test, acute/chronic toxicity test.  7. Dosage of the formula, and how the dosage was determined.  8. Administration route (e.g. oral, external).  For individualized CHM formulae  1. As for fixed CHM formulae, refer to fixed formulae Point 1-8 listed above.  2. Additional information: how, when and by whom the formula will be modified.  For patent proprietary CHM formulae  1. Reference to a publicly available material(s), such as Pharmacopeia, for the details about the composition, dosage, efficacy, safety, and quality control of the formula.  2. Illustration of the details of the formula, namely: i) the proprietary product name (i.e. brand name), ii) name of manufacturer, iii) lot number, iv) production data and expiry date, v) name and content of added materials, and vi) whether any additional quality control procedures will be conducted.  3. Statement of whether the patent proprietary CHM formula utilized in the study is identical to the publicly available reference. | | 8 & supplemental file 1 |
|  | |  |  | 11a.1B. Acupuncture  1. Treatment environment and participant posture.  2. Number of needle insertions per subject per session (mean and range if possible).  3. Names and location of acupoints (uni/bilateral). Name of all acupoints should be presented in Chinese (Pinyin) and international code.  4. Angle and depth of insertion, which should be presented in a specified unit of measurement or on a particular tissue level.  5. Response sought (e.g. de qi or muscle twitch response).  6. Needle stimulation (e.g. manual, electrical). If electroacupuncture apparatus will be utilized, the brand, manufacturer and frequency should be indicated.  7. Needle retention time.  8. Needle type, including diameter, length, manufacturer and material, etc.  9. Number of treatment sessions.  10. Frequency and duration of treatment sessions. | | NA |
|  | |  |  | 11a.1C. Moxibustion  1. Treatment environment and participant posture.  2. Number of moxibustion units per subject per session (mean and range if possible).  3. Names and location of acupoints (uni/bilateral). Name of all acupoints should be presented in Chinese (Pinyin) and international code.  4. Procedure and technique of moxibustion (e.g. direct/indirect, warming/sparrow-pecking technique, warming needle, moxa box).  5. Response sought (e.g. warm feeling, skin reddening, burning pain).  6. Moxibustion retention time.  7. Materials used for moxibustion (e.g. moxa floss, moxa cone, moxa stick, herbal patches, and their sizes and manufacturers).  8. Number of treatment sessions.  9. Frequency and duration of treatment sessions. | | NA |
|  | |  |  | 11a.2 Interventions for the control group(s) with sufficient detail to allow replication. | | 8 |
|  | |  |  | 11a.2A. CHM formulae  Placebo control  1. Name and dosage of each ingredient.  2. Description of the similarity of placebo with intervention (e.g. color, smell, taste, appearance, packing).  3. Quality control and safety surveillance, if any.  4. Administration route, dosage and regimen.  5. Production information: when, where, how and by whom the placebo was produced.  Active control  1. If a CHM formula was used, refer to the recommendations of 11a.1A.  2. If a chemical agent was used, the name, administration route, dosage and regime should be included. | | 8 |
|  | |  |  | 11a.2B. Acupuncture  Blank/waitlist control  State any special arrangement(s) in pre-treatment, treatment and post-treatment periods corresponding to the experimental intervention (e.g. examinations in pre-treatment period, unaltered lifestyle and medication in treatment period, and compensatory interventions in post-treatment period).  Sham acupuncture or acupuncture-like control  State the comparability of the sham acupuncture or acupuncture-like control and comprehensively provide details as for the recommendations of 11a.1B. | | NA |
|  | |  |  | 11a.2C. Moxibustion  Blank/waitlist control  State any special arrangement(s) in pre-treatment, treatment and post-treatment periods corresponding to the experimental intervention (e.g. examinations in pre-treatment period, unaltered lifestyle and medication in treatment period, and compensatory interventions in post-treatment period).  Sham moxibustion or moxibustion-like control  State the comparability of the sham moxibustion or moxibustion-like control and comprehensively provide details as for the recommendations of Intervention 11a.1C. | | NA |
|  | | 11b. Criteria for discontinuing or modifying allocated interventions for a given trial participant (eg, drug dose change in response to harms, participant request, or improving/worsening disease) |  |  | | NA |
|  | | 11c. Strategies to improve adherence to intervention protocols, and any procedures for monitoring adherence (eg, drug tablet return, laboratory tests) |  |  | | 9，11 |
|  | | 11d. Relevant concomitant care and interventions that are permitted or prohibited during the trial |  | 11d.2 Descriptions of other interventions that will be administrated to experimental and/or control groups are recommended (e.g. rescue interventions), with enough details to allow replication. | | 9 |
| Outcomes | | 12. Primary, secondary, and other outcomes, including the specific measurement variable (eg, systolic blood pressure), analysis metric (eg, change from baseline, final value, time to event), method of aggregation (eg, median, proportion), and time point for each outcome. Explanation of the clinical relevance of chosen efficacy and harm outcomes is strongly recommended |  | 12a. Provide the rationale of TCM-related indexes as outcomes (e.g. the change of degree and scope of symptoms and signs related to Pattern differentiation). | | 9-11 |
|  |  |  |  | 12b. Provide the details of the TCM-related outcomes assessment, including i) the measuring methods and standard (e.g. frequency, severity rating scale of symptoms and signs, verified Pattern questionnaire, time points for assessment and corresponding rationale), ii) assessor qualification (e.g. relevant assessment experience, years in clinical practice), iii) methods used to enhance the quality of assessment (e.g. multiple repeated observation, training of assessors), and iv) related reference(s). | |  |
| Participant timeline | | 13. Time schedule of enrolment, interventions (including any run-ins and washouts), assessments, and visits for participants. A schematic diagram is highly recommended |  |  | | 9-11 |
| Sample size | | 14. Estimated number of participants needed to achieve study objectives and how it was determined, including clinical and statistical assumptions supporting any sample size calculations |  |  | | 12 |
| Recruitment | | 15. Strategies for achieving adequate participant enrolment to reach target sample size |  |  | | 5 |
| **Assignment of interventions (for controlled trials)** | | | | | | |
| Sequence generation | | 16a. Method of generating the allocation sequence (eg, computer-generated random numbers) and list of any factors for stratification. To reduce predictability of a random sequence, details of any planned restriction (eg, blocking) should be provided in a separate document that is unavailable to those who enroll participants or assign interventions |  |  | | 7 |
| Allocation concealment mechanism | | 16b. Mechanism of implementing the allocation sequence (eg, central telephone; sequentially numbered, opaque, sealed envelopes), describing any steps to conceal the sequence until interventions are assigned |  |  | | 7 |
| Implementation | | 16c. Who will generate the allocation sequence, who will enroll participants, and who will assign participants to interventions |  |  | | 7 |
| Blinding (masking) | | 17a. Who will be blinded after assignment to interventions (eg, trial participants, care providers, outcome assessors, data analysts) and how |  |  | | 7-8 |
|  | | 17b. If blinded, circumstances under which unblinding is permissible and procedure for revealing a participant’s allocated intervention during the trial |  |  | | 7-8 |
| **Data collection, management, and analysis** | | | | | | |
| Data collection methods | | 18a. Plans for assessment and collection of outcome, baseline, and other trial data, including any related processes to promote data quality (eg, duplicate measurements, training of assessors) and a description of study instruments (eg, questionnaires, laboratory tests) along with their reliability and validity, if known. Reference to where data collection forms can be found, if not in the protocol |  | 18a. When trial targeting on TCM Pattern, or a WM-defined disease with a specific TCM Pattern, baseline data about TCM Pattern should be provided. | | 9-10 |
|  | | 18b. Plans to promote participant retention and complete follow-up, including list of any outcome data to be collected for participants who discontinue or deviate from intervention protocols |  |  | | NA |
| Data management | | 19. Plans for data entry, coding, security, and storage, including any related processes to promote data quality (eg, double data entry; range checks for data values). Reference to where details of data management procedures can be found, if not in the protocol |  |  | | 13 |
| Statistical methods | | 20a. Statistical methods for analysing primary and secondary outcomes. Reference to where other details of the statistical analysis plan can be found, if not in the protocol |  |  | | 12-13 |
|  | | 20b. Methods for any additional analyses (eg, subgroup and adjusted analyses) |  |  | | 12-13 |
|  | | 20c. Definition of analysis population relating to protocol non-adherence (eg, as randomised analysis), and any statistical methods to handle missing data (eg, multiple imputation) |  |  | | 12-13 |
| **Monitoring** | | | | | | |
| Data monitoring | | 21a. Composition of data monitoring committee (DMC); summary of its role and reporting structure; statement of whether it is independent from the sponsor and competing interests; and reference to where further details about its charter can be found, if not in the protocol. Alternatively, an explanation of why a DMC is not needed |  |  | | 13 |
|  | | 21b. Description of any interim analyses and stopping guidelines, including who will have access to these interim results and make the final decision to terminate the trial |  |  | | NA |
| Harms | | 22. Plans for collecting, assessing, reporting, and managing solicited and spontaneously reported adverse events and other unintended effects of trial interventions or trial conduct |  |  | | 11 |
| Auditing | | 23. Frequency and procedures for auditing trial conduct, if any, and whether the process will be independent from investigators and the sponsor |  |  | | 13 |
| **Ethics and dissemination** | | | | | | |
| Research ethics approval | | 24. Plans for seeking research ethics committee/institutional review board (REC/IRB) approval |  |  | 13 | |
| Protocol amendments | | 25. Plans for communicating important protocol modifications (eg, changes to eligibility criteria, outcomes, analyses) to relevant parties (eg, investigators, REC/IRBs, trial participants, trial registries, journals, regulators) |  |  | NA | |
| Consent or assent | | 26a. Who will obtain informed consent or assent from potential trial participants or authorised surrogates, and how (see Item 32) |  |  | 13 | |
|  | | 26b. Additional consent provisions for collection and use of participant data and biological specimens in ancillary studies, if applicable |  |  | NA | |
| Confidentiality | | 27. How personal information about potential and enrolled participants will be collected, shared, and maintained in order to protect confidentiality before, during, and after the trial |  |  | NA (In this stage, no data is collected.) | |
| Declaration of interests | | 28. Financial and other competing interests for principal investigators for the overall trial and each study site |  |  | 15 | |
| Access to data | | 29. Statement of who will have access to the final trial dataset, and disclosure of contractual agreements that limit such access for investigators |  |  | 16 | |
| Ancillary and post-trial care | | 30. Provisions, if any, for ancillary and post-trial care, and for compensation to those who suffer harm from trial participation |  |  | NA | |
| Dissemination policy | | 31a. Plans for investigators and sponsor to communicate trial results to participants, healthcare professionals, the public, and other relevant groups (eg, via publication, reporting in results databases, or other data sharing arrangements), including any publication restrictions |  | Plan for raw data sharing, if any. The contents should contain: i) when the data will become available; ii) how the data will be shared iii) what data in particular will be shared; iv) who could acquire the data; v) through what access data will be shared. | 16 | |
|  | | 31b. Authorship eligibility guidelines and any intended use of professional writers |  |  | NA | |
|  | | 31c. Plans, if any, for granting public access to the full protocol, participant level dataset, and statistical code |  |  | NA | |
| **Appendices** | | | | | | |
| Informed consent materials | | 32. Model consent form and other related documentation given to participants and authorised surrogates |  |  | | Supplemental file |
| Biological specimens | | 33. Plans for collection, laboratory evaluation, and storage of biological specimens for genetic or molecular analysis in the current trial and for future use in ancillary studies, if applicable |  |  | | N |
