# Supplementary material for: Chinese Herbal Formula Xuefu Zhuyu for Tension-Type Headache with Qi-Stagnation and Blood-Stasis Pattern (CheruXTH): Study Protocol for a Randomized Controlled Trial
Source: Evid Based Complement Alternat Med. 2020 Sep 11;2020:5653169. doi: 10.1155/2020/5653169 (PMC7502128; doi:10.1155/2020/5653169)
Supplement: Supplementary Materials — S1: the dose and scientific names of all ingredients in XFZY oral liquid. S2: SPIRIT-TCM Extension 2018 checklist. [file 5653169.f1.zip › 5653169.f1/supplementary file 1--XFZY dose and differerent names and pharmaceutical production processes.docx]

Supplemental file 1

The dose and scientific names of all ingredients in XFZY oral liquid

| **Chinese name** | **English name** | **Latin name** | **Dose*** |
| --- | --- | --- | --- |
| Chaihu | Bupleuri Radix | *Bupleurum chinense DC.* | 17g |
| Danggui | Angelicae Sinensis Radix | *Angelica sinensis (Oliv.) Diels* | 50g |
| Shengdihuang | Rehmanniae Radix | *Rehmannia glutinosa Libosch.* | 50g |
| Chishao | Paeoniae Radix Rubra | *Paeonia lactiflora Pall* | 33g |
| Honghua | Carthami Flos | *Carthamus tinctorius L.* | 50g |
| Taoren | Persicae Semen | *Prunus persica (L.)Batsch* | 67g |
| Zhiqiao | Aurantii Fruxtus | *Citrus aurantium L.* | 33g |
| Gancao | Glycyrrhizae Radix et Rhizoma | *Glycyrrhiza uralensis Fisch.* | 17g |
| Chuanxiong | Chuangxiong Rhizoma | *Ligusticum chuanxiong Hort.* | 25g |
| Niuxi | Achyranthis Bidentatae Radix | *Achyranthes bidentata Bl.* | 50g |
| Jiegeng | Platycodonis Radix | *Platycodon grandiflorum (Jacq.) A.DC.* | 25g |

*The dose of each drug in 1000ml liquid

Pharmaceutical production processes:

XFZY oral liquid was manufactured in strict accordance with the standards of the Chinese Pharmacopoeia (2015), and the process strictly abides by the good manufacturing practices (GMP). The outline is as following:

There are 11 herbal medicines in the liquid, Bupleuri Radix, Angelicae Sinensis Radix, Aurantii Fruxtus, and Chuangxiong Rhizoma distilled to extract aromatic water, set aside; medicine residue and other herbal medicines were decocted 3 times, 2 hours each time, combine decoction, filter, and filtrate concentrated to a relative density of about 1.10 (60°C), add ethanol to make the alcohol content up to 60%, refrigerate for 24 hours, filter, and recycle the filtrate to the ethanol until no alcohol taste, add 100 g sucrose, 200 g honey, 0.5 g potassium sorbate and the above aroma water, stir, add water to 1000 ml and mix well, adjust the pH to 5.0, refrigerate, filter, fill, sterilize, and get ready
